# Evaluation of the Antioxidant Activity of *Cis*/*Trans*-*N*-Phenyl-1,4,4a,5,8,8a-Hexahydro-3,1-Benzoxazin-2-Imines

**DOI:** 10.3390/antiox8060197

**Published:** 2019-06-25

**Authors:** Guadalupe Firpo, María L. Ramírez, Martín S. Faillace, Maria dos R. Mendes de Brito, Ana P. S. Correia Lima e Silva, Jessica Pereira Costa, Marcela C. Rodríguez, Gustavo A. Argüello, Zsolt Szakonyi, Ferenc Fülöp, Walter J. Peláez

**Affiliations:** 1 INFIQC-CONICET. Departamento de Físicoquímica, Facultad de Ciencias Químicas, Universidad Nacional de Córdoba, X5000HUA Córdoba, Argentina; gfirpo@fcq.unc.edu.ar (G.F.); mramirez@fcq.unc.edu.ar (M.L.R.); mfaillace@unc.edu.ar (M.S.F.); marcela.rodriguez@fcq.unc.edu.ar (M.C.R.); 2 Department of Pharmaceutical Chemistry, Faculty of Facid Wyden, Federal University of Piauí, Teresina, PI. CEP 64.052-410, Brazil; mbrito@facid.edu.br (M.d.R.M.d.B.); jessicaprcosta@ufpi.edu.br (J.P.C.); gaac@fcq.unc.edu.ar (G.A.A.); 3 Department of Pharmaceutical Chemistry, Faculty of Nursing, Federal University of Piauí, Teresina, PI. CEP 64.049-550, Brazil; apaulasantoslima@ufpi.edu.br; 4 Institute of Pharmaceutical Chemistry, University of Szeged, H-6720 Szeged, Eötvös utca 6, Hungary; fulop@pharm.u-szeged.hu

**Keywords:** antioxidant activity, unsaturated-fused 1,3-oxazines, ET mechanism, antiradical power

## Abstract

The growing interest in the chemistry of unsaturated ring-fused 1,3-heterocycles, in this particular case 1,3-oxazines, arise in part from their versatile pharmacological applications. In the present article, the evaluation of the in vitro and ex vivo antioxidant activity of two cyclohexene-fused oxazines is discussed. The in vitro antioxidant activity was evaluated by trapping the ABTS and hydroxyl radicals as well as the inhibition of the enzyme acetyl-cholinesterase and hemolysis of erythrocytes by 2,2’-Azobis(2-amidinopropane) dihydrochloride (AAPH). The results suggest that both unsaturated 1,3-oxazines are auspicious sources of biologically active compounds with good antioxidant properties. In addition, a comprehensive analysis of the interaction between these heterocycles with 2,2-diphenyl-1-picrylhydrazyl (DPPH) and 2,2’-azino-bis(3-ethylbenzothiazoline-6-sulphonic acid) (ABTS) radicals, as well as the measurements of redox potential, provided evidence for a mechanism of antioxidant activity that takes place through electron transfer (ET) processes.

## 1. Introduction

Free radicals are responsible for intercellular signaling and synthesis of important biological substances in living organisms, due to their ability to facilitate the donation of their electrons to other molecules, which causes chain reactions [1,2]. However, some free radicals, called reactive oxygen species (ROSs), as well as other radical promoters, are extremely harmful leading to deleterious effects such as DNA, proteins and cell organelles damage [3,4,5,6,7]. ROSs such as superoxide (O_2_^●^), hydroxyl (OH^●^) and hydrogen peroxide (H_2_O_2_) are generated by the oxygen metabolism pathway [8].

Some physiological processes are responsible for the antioxidant defense mechanism that catalyzes the reduction of O_2_ in water. Such processes control the generation of free radicals and prevent their overproduction in mitochondria [9]. However, there are some diseases where oxidative stress is involved in the pathophysiology, and often, the defense mechanism is not enough. In these cases, it is necessary to find new antioxidant compounds as therapeutic agents [10].

Studies conducted with synthetic compounds have shown good results in the field of antioxidant capacity. Among these groups of substances, oxazines have played an important role due to their wide pharmacological applications. In this sense, the derivative 2-ethoxy-4,5-diphenyl-1,3-oxazine-6-one exhibit a neuroprotective effect in rat pheochromocytoma cells against cellular death induced by H_2_O_2_ [11]. Similarly, 1,3-oxazine substituted 9-anilinoacridine derivatives [12] and bi- and tetra-substituted dihydro-1,3-oxazines [13] were evaluated for their antioxidant activities. In addition, the derivative tetrahydro-1,4-oxazine is known to be biologically active against enzymes that metabolize inflammatory drugs [2]. 

**Figure 1:**
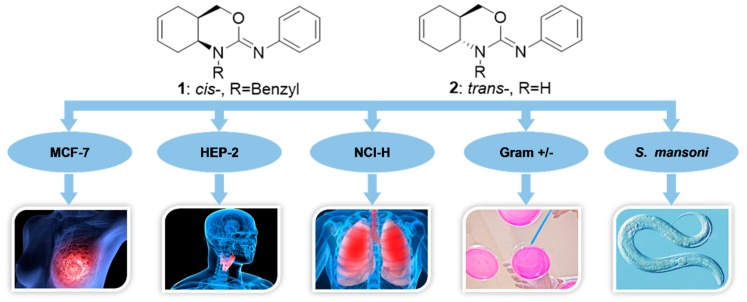
cis-1-benzyl-*N*-phenyl-1,4,4a,5,8,8a-hexahydro-2*H*-benzo[d][1,3]oxazin-2-imine (**1**) and trans-*N*-phenyl-1,4,4a,5,8,8a-hexahydro-2*H*-benzo[d][1,3]oxazin-2-imine (**2**) prepared for this study and their different biological activities previously described. MCF-7: Michigan Cancer Foundation, type 7 human breast carcinoma cells; HEP-2: Human epithelial, type 2 human larynx carcinoma cells; NCI-H: National Cancer Institute, human lung carcinoma cells; *S. mansoni*: *Schistosoma mansoni*.

As part of our ongoing research on partially saturated 1,3-heterocycles [14,15,16,17,18], we have published the synthesis of cyclohexene-fused 1,3-oxazines with selective antibacterial and antiparasitic action along with low cytotoxic effects (compounds **1**, **2**, Figure 1) [19].

Finding new compounds with antioxidant activity is of utmost importance, this new study aims to evaluate the antioxidant potential of these synthetic oxazines through in vitro and ex vivo methodologies. The deep interest in the antioxidant behavior of these compounds arises in part from their versatile synthetic applicability and their potential pharmacological applications [15,20,21,22,23,24,25].

In order to measure antioxidant activity, different techniques and procedures have been proposed. Depending on the reactions involved, in vitro assays can roughly be classified into two types: assays based on hydrogen atom transfer (HAT) and assays based on electron transfer (ET). HAT-based assays measure the capability of an antioxidant to quench free radicals by H-atom donation. On the other hand, ET-based assays measure the capacity of an antioxidant in the reduction of an oxidant, which changes color when reduced [26]. The results obtained by HAT and ET based assays are hardly comparable because of the different driving mechanisms, redox potentials, pH and solvent dependencies between them [27]. Other types of antioxidant assays are based on lipids oxidation, which is a key issue mainly in food deterioration and oxidative modification of low-density lipoprotein (LDL) [28]. Some examples of these assays are the hemolysis potential and thiobarbituric acid reactive substances (TBARS) analysis.

In this work, in vitro antioxidant properties of oxazines (**1**,**2**) were evaluated by inhibition of 2,2’-azino-bis(3-ethylbenzthiazoline-6-sulfonic acid) (ABTS^●+^), OH^●^, 2,2-diphenyl-1-picrylhydrazyl (DPPH^●^) and the enzyme acetylcholinesterase (AChE). In addition, through electrochemical assays, oxidation redox potential was also measured. At the same time, control substances like trolox (Tx), ascorbic acid (AAc) and hydroquinone sulfonic acid (HQSAc) were also tested and used as the simplest target to elucidate the possible mechanisms occurring and to determine the antiradical power (ARP) along with the stoichiometry of the reactions. Furthermore, ex vivo antioxidant activity was measured by testing the hemolysis inhibition of rat erythrocytes after induction by 2,2’-azo-bis(amidinopropane) dihydrochloride (AAPH).

## 2. Materials and Methods

### 2.1. Evaluation of in Vitro Antioxidant Potential by Inhibition of ABTS^●+^ Radical

The methodology described by Re et al. [29] was used for the determination of the antioxidant capacity by the ABTS^●+^ method. Initially, the ABTS^●+^ radical cation was formed from the reaction of 2,2’-azino-bis(3-ethylbenzothyazoline-6-sulfonic acid (ABTS in water, 5 mL, 7 mM) with potassium persulfate (K_2_S_2_O_8_ in water, 88 µL, 2.45 mM) and incubated in darkness for 16 h at room temperature. Then, the solution of ABTS^●+^ was diluted in ethanol to obtain an absorbance of 0.70 ± 0.05 at 734 nm and 1960 µL were fed in each of ten test tubes keeping the dark conditions. Different aliquots of oxazines 1 and 2 were added to this solution to reach final concentrations of 9.0, 13.5, 18.0, 22.5 and 27.0 µg/mL and after 6 min, the absorbance was measured at 734 nm. The results, expressed as percentages of ABTS^●+^ radical inhibition were taken from Equation (1), and from their values, the effective inhibitory concentration (EC_50_) at 734 nm was determined. The same experimental procedure was used with the standard positive control Tx.

% inhibition of ABTS^●+^ = {(A_control_ − A_reaction mixture_) × 100}/A_control_ (1)

A_control_ is the initial absorbance of the ethanol solution of ABTS^●+^.

### 2.2. Evaluation of In Vitro Antioxidant Potential Measured against the Production of Hydroxyl Radical

For the evaluation of the antioxidant potential, measured against the production of OH^●^ generated by the Fenton reaction, the methodology described by Lopes et al. [30] with some modifications was used. Different aliquots of oxazines 1 and 2 were added to 300 µL of Fenton reaction mixture, which contains FeSO_4_ (6 mM), 2-deoxyribose (5 mM), H_2_O_2_ (100 mM) and phosphate buffer (20 mM, pH 7.4) in order to reach final concentrations of 9.0, 13.5, 18.0, 22.5 and 27.0 µg/mL. The reaction was allowed to proceed for 15 min at room temperature and then 4% phosphoric acid followed by 1% thiobarbituric acid (TBA in NaOH 50 mM) were added. Then, the mixture was heated for 1 h at 37 °C and finally cooled down to room temperature. The experiment was performed by triplicate and the absorbance readings measured at 532 nm. The results were expressed as a percentage of 2-deoxyribose degradation. The effective inhibitory concentration of the compound required to inhibit the degradation of 2-deoxyribose by 50% (EC_50_) was determined at 532 nm. The same experimental procedure was also used with the positive control Tx at the same set of concentrations.

### 2.3. Evaluation of In Vitro Antioxidant Potential by Inhibition of DPPH Radical

The antioxidant activities of different compounds against DPPH^●^ were performed following the procedures described by Brand-Williams et al. [31]. Different concentrations of each tested compound in ethanol (0.1 mL), were added dropwise to 3.9 mL of DPPH^●^ solution (6×10^−5^ mol L^−1^ in ethanol) and the absorbance decay was measured at 517 nm at *t* = 0, 1 and every 15 min until the reaction reached a plateau. The initial DPPH^●^ concentration was determined by a calibration curve. For each antioxidant concentration tested, the percentage remaining of DPPH^●^ was plotted against time. From these plots, the percentage of DPPH^●^ at the steady state (at each antioxidant concentration) was determined. These values were transferred onto another graph and plotted as a function of the molar ratio of antioxidant to DPPH^●^ (moles antioxidant/mole DPPH^●^) to determine, the rEC_50_ which is the relative amount necessary to decrease the initial DPPH^●^ concentration to 50%.

### 2.4. Evaluation of the Qualitative Inhibition of the Enzyme Acetylcholinesterase

The inhibitory activity of the enzyme was controlled using the methodology proposed by Rhee et al. [32], who adapted the procedure established by Ellman et al. [33]. Initially, the compounds to be analyzed were dissolved in methanol and then spotted on a thin layer chromatography (TLC) plate. The compounds were eluted with a mixture of chloroform and methanol (9:1). Then, the plate was sprayed with 5,5’-dithio-bis[2-nitrobenzoic acid] (DTNB) and acetylthiocholine iodide in Tris-HCl buffer (pH 8). Five minutes later, the enzyme AChE was sprayed and the inhibition was observed. For these experiments, caffeine was used as a positive standard [34].

### 2.5. Test of Hemolysis Inhibition in Erythrocytes of Rattus norvegicus Induced by AAPH

To evaluate the antioxidant capacity by hemolysis inhibition of rat erythrocytes (*R*. norvegicus, Wistar type), the methodology described by Ugartondo et al. [35] with some modifications was used. Animal care followed the official governmental guidelines in compliance with the Society Policy and was submitted by the Ethics Committee of the Federal University of Piauí [36]. Briefly, an erythrocyte suspension (200 µL) and a solution of AAPH (100 µL, 20 nM) in PBS (Phosphate Buffered Saline, MERK KGaA, Darmstadt, Germany, pH 7.4), were mixed with oxazines 1 or 2 in order to reach a final concentration of 100 µg/mL. Simultaneously, a control was set to detect the spontaneous hemolysis. The reaction mixture was incubated at 37 °C for 30 min followed by a centrifugation step performed at 2500 rpm for 5 min. Then, PBS (800 µL) was added to the supernatant obtained (200 µL) in order to determine hemolysis by measuring the absorbance at 540 nm. The same experimental procedure was also used with the standard positive control Tx.

The results were expressed as percentage of hemolysis inhibition using an equation similar to Equation (1), where the A_control_ corresponds now to the hemolysis absorbance of 2% erythrocytes suspension induced by AAPH (100% hemolysis). The erythrocytes suspension in PBS was taken as 0%.

### 2.6. Determination of Redox Potential

Certainly, one of the most powerful, simple and frequently used electrochemical techniques (often along with DPPH^●^ assay) for the determination of the antioxidant activity is cyclic voltammetry (CV) [37,38]. The experimental procedure consists in applying a potential scan from a starting value to a final one, and then returning to the initial potential, while the current flowing between working and counter electrodes is being registered. The potential at which the current increases shows a signal with a peak shape, which corresponds to the oxidation or reduction process of a species in solution. In this way, the oxidation potential can be taken as a measure of the easiness of the oxidation process. Thus, low values of oxidation potentials of a given compound are associated with a greater facility for electro-donation and, therefore, antioxidant activity of such compound [39,40].

Electrochemical experiments were performed with a TEQ_04 potentiostat. Glassy carbon electrodes (3 mm diameter, CH Instruments, Inc. Austin, TX USA) were used as working electrodes. A platinum wire and Ag/AgCl, 3 M NaCl (BAS, Model RE-5B, BASi® Corporate Headquarters, West Lafayette, IN, USA) served as counter and reference electrodes, respectively. All potentials were referred to the later. The electrodes were inserted into a conventional electrochemical cell through holes in its Teflon cover. When the experiment required it, the solution was deoxygenated with ultrapure N_2_. For the subsequent analysis, OriginPro 8.0 software (OriginPro 8.0, OriginLab Corporation, Northampton, MA, USA) was used.

### 2.7. Statistical Analysis

The results, that follow a parametric distribution, were evaluated by analysis of variance (ANOVA) and the Student-Newman-Keuls test as post hoc test. For nonparametric data (percentages), the chi-square test was used (GraphPad PRISM^®^ 6.0 software, GraphPad Software Inc, San Diego, CA, USA). The differences were considered statistically significant for *p* < 0.05. Also, the EC_50_ values and its confidence intervals (95%) for the experiments were obtained using the same GraphPad Prism software.

## 3. Results

### 3.1. In Vitro Antioxidant Potential against the Production of Hydroxyl Radical

**Figure 2:**
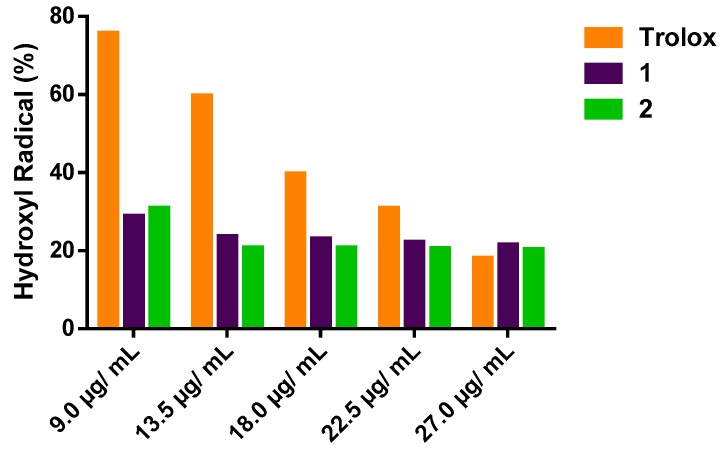
Percentage of remaining hydroxyl radical (OH^●^).

The effect of 1 and 2 on the inhibition of OH^●^ is shown in Figure 2. Both oxazines were able to act as inhibitors of the degradation of 2-deoxyribose by removing significantly the amount of hydroxyl radical (*p* < 0.05) compared to the positive control Tx, (Figure 2). In the concentration range of 9.0 to 27.0 µg/mL, compound 1 showed antioxidant capacity values from 71 to 78.5%, while the antioxidant capacity of 2 ranged from 69.1 to 79.6%. Nevertheless, the positive control showed antioxidant capacity from 24 to 82%, as seen in Table 1. Upon these results for inhibition of the degradation of 2-deoxyribose, the EC_50_ values calculated with the statistical treatment were 0.48 µg/mL, 1.28 µg/mL and 15.21 µg/mL for compounds **1**, **2** and Tx, respectively.

### 3.2. In Vitro Antioxidant Potential by Inhibition of ABTS^●+^ Radical

**Figure 3:**
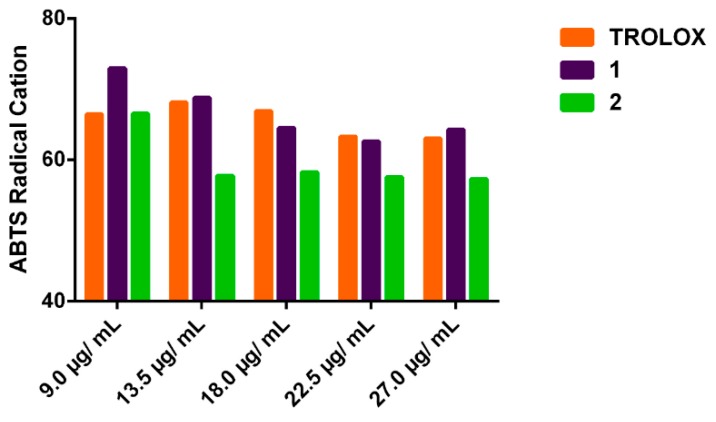
Percentage of ABTS^●+^ remaining.

The antioxidant results related to the capture of ABTS^●+^ radical by 1 and 2 at different concentrations are shown in Figure 3. The values of antioxidant capacity of both oxazines, at the same concentrations employed for the OH^●^ tests, were in the range of 32.0 to 40.8% and 38 to 46.6%, respectively, (Table 2). The oxazine 2 significantly reduced (*p* < 0.05) the concentration of ABTS^●+^ compared to the positive control Tx. In addition, Tx showed antioxidant capacity range value of 36.5 to 41.3%, at the same concentration of 1 and 2. Based on these results, the EC_50_ values (and rEC_50_ calculated as moles of antioxidant/mole ABTS^●+^) were 66.5 µg/mL (0.45), 36.0 µg/mL (0.34) and 34.3 µg/mL (0.30) for oxazine 1, 2 and Tx respectively. In the same order the ARP values calculated as 1/rEC_50_, were 2.2, 2.9 and 3.3.

### 3.3. Hemolysis Inhibition in Erythrocytes of Rattus norvegicus Induced by AAPH and Qualitative Inhibition of the Enzyme Acetylcholinesterase

**Figure 4:**
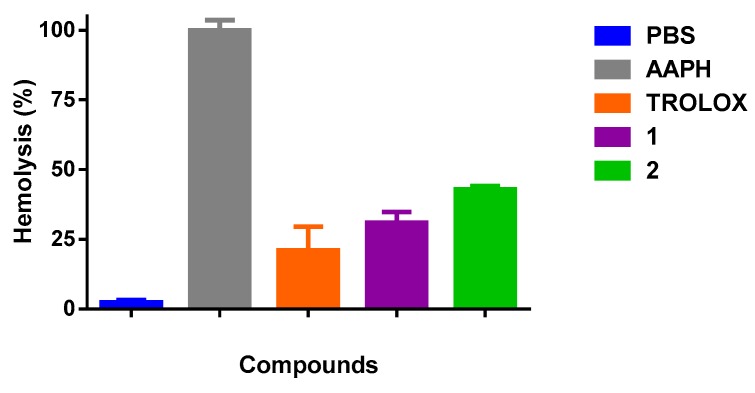
Percentage of hemolysis inhibition induced by 2,2’-Azobis(2-amidinopropane) dihydrochloride (AAPH) in erythrocytes of *Rattus norvegicus.* PBS: Phosphate Buffered Saline.

The results of hemolysis inhibition induced by AAPH (20 nM) in an erythrocytes suspension in the presence of 1 and 2 is shown in Figure 4. The values of the antioxidant activity at the concentration of 100 µg/mL were (69 ± 2%, *p* < 0.05) and (57.1 ± 0.6%, *p* < 0.05), respectively. As it is possible to see, both oxazines decrease significantly the hemolysis effect compared to AAPH. Tx at 100 µg/mL, also reduced the percentage of hemolysis significantly (79 ± 5%, *p* < 0.05) compared to AAPH. Finally, as indicated in the experimental section, we also tested the qualitative inhibition of the enzyme AChE with the oxazines, obtaining in both cases positive results.

### 3.4. Cyclic Voltammetry

**Figure 5:**
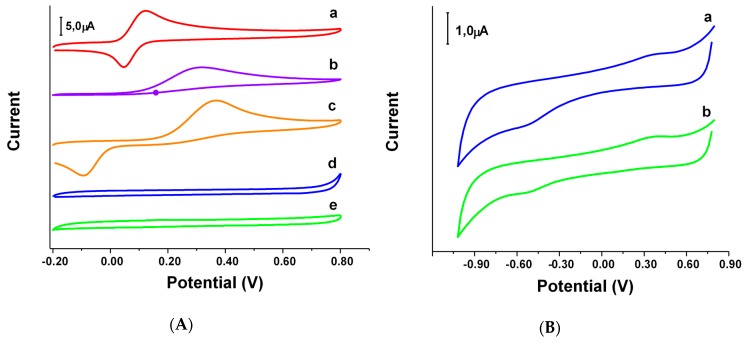
(**A**) Cyclic voltammograms for 5.0 × 10^−4^ M (a) Tx, (b) AAc, (c) HQSAc, and 1.0 × 10^−3^ M (d) oxazine 1 and (e) oxazine 2 at a bare glassy carbon electrodes (GCE); (**B**) Cyclic voltammograms for 1.0 × 10^−3^ M (a) oxazine 1 and (b) oxazine 2 in a deoxygenated solution at a bare GCE. Supporting electrolyte: 0.050 M phosphate buffer solution pH 7.40. Scan rate: 0.100 V s^−1^.

The redox behavior of the synthesized oxazines 1 and 2 was evaluated using cyclic voltammetry (CV) (Figure 5), recording the voltammograms in buffer phosphate 0.050 M pH 7.40 and comparing the electrochemical behavior of oxazines 1 and 2 to that of reference compounds Tx, AAc and HQSAc. These substances are well-known for either having antioxidant activity (Tx and AAc) or a structure that some authors considered as potentially good antioxidants (HQSAc) [41]. In this sense, oxazines 1 and 2, due to their chemical structure, could be classified into the last group. For a better analysis, the oxidation and reduction peak potentials (denoted as E_ap_ and E_cp_, respectively) and the difference (∆E_p_) expressed as the absolute value of the difference between E_cp_ and E_ap_, were evaluated as well. In Figure 5A it is possible to see that the voltammetric responses for Tx, AAc and HQSAc show well-defined redox profiles. As it was expected, the lowest E_ap_ is observed for Tx (0.123V), being 0.322 V and 0.367 V the results obtained for AAc and HQSAc, respectively. However, for oxazine 1 and 2 the redox processes are negligible, even at higher concentrations, which suggest that the electron transfer processes of these compounds are certainly impeded. In order to dilucidate the redox behavior of oxazines 1 and 2, it was necessary to set a new wider potential window from −1.20 V to 0.90 V. Under these new conditions, the characteristic redox process of oxygen could be an important interference in the determination of the reduction potentials of oxazine 1 and 2. Therefore, the deoxygenation of the solution was required. As it can be seen in Figure 5B, the oxidation potentials for oxazine 1 and 2 were 0.420 and 0.380, respectively.

The values of ∆E_p_ were obtained in separated experiments for each compound and performed in a three-electrode conventional electrochemical cell using a glassy carbon working electrode (other conditions as detailed in experimental section). The ∆E_p_ found values were 0.076 V and 0.273 V for Tx and HQSAc, respectively (Figure 5A). The ∆E_p_ value for AAc could not be calculated due to the existence of a unique peak for the oxidation process because of its irreversible behavior under the tested conditions. The ∆E_p_ values for oxazine 1 and 2 where 0.979 V and 0.939 V, respectively (Figure 5B). These results indicate that the glassy carbon working electrode surface is less reactive for the heterogeneous redox processes of oxazine 1 as well as 2 presenting a kind of limitation in comparison with the reference compounds.

### 3.5. In Vitro Antioxidant Potential by Inhibition of DPPH^●^ Radical

**Figure 6:**
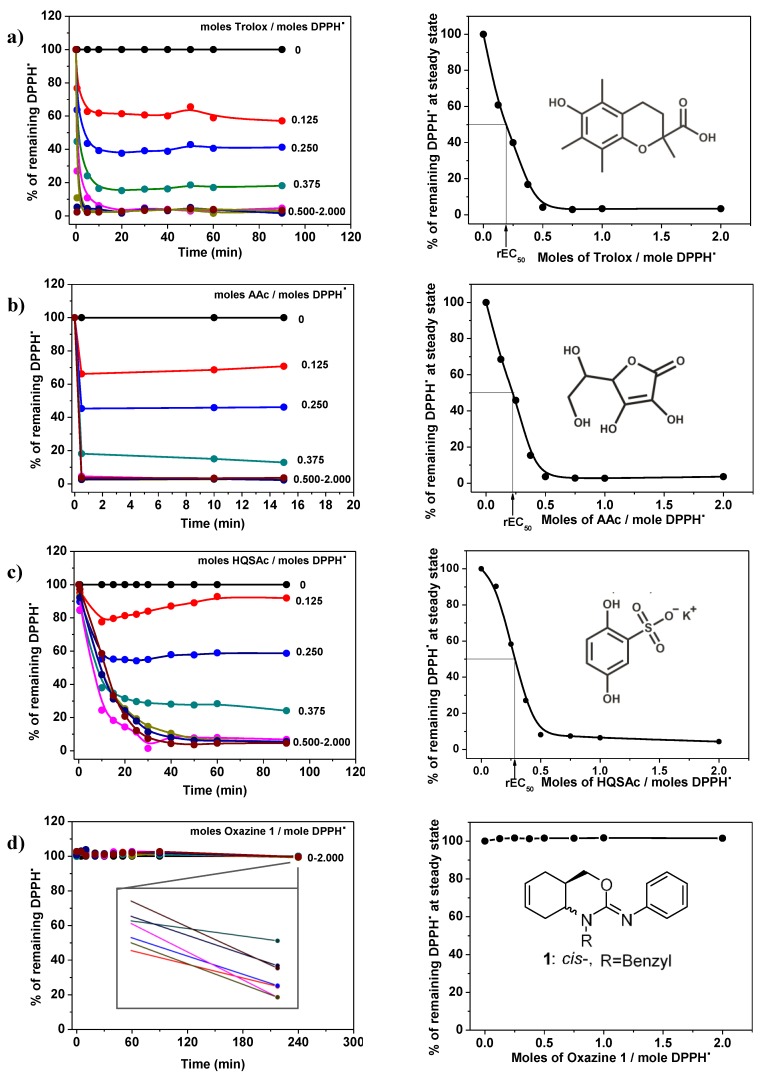
Reaction respond curves and rEC_50_ determined by inhibition of 2,2-diphenyl-1-picrylhydrazyl (DPPH) radical. (**a**) Trolox; (**b**) AAc; (**c**) HQSAc and (**d**) Oxazine **1**.

The antioxidant abilities were determined using DPPH^●^ as a free radical. For each antioxidant, different concentrations were tested (expressed as the number of moles of antioxidant/mole DPPH^●^) and the reaction response curves were plotted, (Figure 6, left). From these results, the relative efficient concentration (rEC_50_) and the antiradical power (ARP = 1/rEC_50_) were determined: the increase of the ARP infers a higher efficient antioxidant ability, (Figure 6, right).

As expected, Tx and AAc have a very good response reaching a steady state in less than 2 min and their ARPs were 4.9 and 4.4, (Figure 6a,b, respectively). On the other hand, HQSAc shows an intermediate behavior, where the steady state was reached after approximately 20 min and its ARP was 3.6, (Figure 6c). Apparently, oxazines 1 and 2 do not react with DPPH^●^ after 4 h, as it is possible to see in Figure 6d for oxazine 1. Nevertheless, it has been reported that some compounds, like guaiacol and its derivatives, have delayed responses in these kinds of assays, taking approximately 6 h to reach the steady state [31]. From a deeper analysis of the oxazine 1 reaction response plots (local enlargement in Figure 6d), it is possible to observe that it reacts very slowly, demonstrating a negligible antioxidant ability, in spite of the results obtained from the other tests performed in this work. A similar behavior could be observed from the analysis of 2 (data not shown).

## 4. Discussion

The biology of free radicals is an emerging area, which mainly explores the formation and elimination of these harmful compounds, as well as the damage they produce on biological systems. It is established that a number of radicals centered in free oxygen and other ROSs contribute to the pathology of many disorders, including atherogenesis, neurodegeneration, chronic inflammation [42,43], cancer and physiological senescence [44]. Due to this, there is an increasing interest in antioxidant processes as a way to aid in the treatment of diseases associated with free radicals.

The OH^●^ radical is one of the most deleterious free radicals because its half-life is very short and can hardly be captured in vivo. This radical often attacks molecules through hydrogen abstraction and it can be generated by reaction of H_2_O_2_ with transition metals or through homolysis of water by exposure to ionizing radiation [4]. The in vitro antioxidant capacity of 1 and 2 against the production of hydroxyl radical is demonstrated by the Fenton reaction. Both oxazines gave remarkable results, i.e., 1, EC_50_ = 0.48 µg/mL and 2, EC_50_ = 1.28 µg/mL compared with the standard Tx (EC_50_ = 15.21 µg/mL). It is important to highlight that even at the lowest dose of oxazines tested (9.0 µg/mL), they showed a higher reduction of radicals than the standard Tx (9.0–22.5 µg/mL).

The in vitro antioxidant capacity was also determined by studying the trapping of ABTS^●+^. This study demonstrated that 1 and 2 are efficient antioxidants for the effective removal of the ABTS^●+^ radical. It is important to note, that both heterocycles showed good antioxidant capacity, showing oxazine 2 an ARP (2.9) similar to Tx (3.3). The antioxidant capacity of these two substances was proportional to the concentration.

The inhibition assay of the AchE was qualitatively demonstrated. This fact is visibly revealed by the color change of the sample from yellow to white in the chromatographic plate. The achieved results are in accordance with Sukhorukov’s observation [2], who demonstrated that 1,3-oxazines, representatives of the library that they prepared, were capable to inhibit the activity of the enzyme AchE.

Alternatively, the in vitro antioxidant capacity was also explored for possible ex vivo protection of biomolecules from damage caused by free radicals [45]. For this evaluation, oxazines 1 and 2 were tested ex vivo using erythrocyte of *Rattus norvegicus*. The erythrocytes are particularly susceptible to oxidative injury due to the high polyunsaturated fatty acid content of their membranes and the high cellular oxygen and hemoglobin (Hb) concentrations. Exposure of erythrocytes to free radicals lead to damage of cell membrane (lipid peroxidation), changes in cellular morphology, protein cross-linking, and sulfhydryl group oxidation, which can subsequently result in membrane damage and hemolysis [46]. Therefore, because of its susceptibility to oxidation, erythrocytes have been used as cell models to investigate oxidative damage on biomembranes [47]. According to the results described above, oxazines 1 and 2 were able to protect erythrocytes from damage induced by AAPH, since there was a decrease in the hemolysis of rat erythrocytes.

On the other hand, two complementary assays were employed to shed light about the mechanism operating in the antioxidant ability of the studied compounds (Table 3). It is well known that ABTS^●^^+^ tests go through an ET process while DPPH^●^ assay was believed to only involve HAT mechanism. However, evidence provided by experts in the area [48,49,50], have amply demonstrated that DPPH^●^ can be quenched by both electron and atom transfer. As it was expected, analysis of the results shows that Tx possesses the highest antioxidant activity/capacity (ARP = 4.9 and 3.3 for DPPH^●^ and ABTS^●+^ determinations, respectively; entry 1 Table 3). In the same way, ARP values obtained for oxazines **1** and **2** by the ABTS^●^^+^ test were slightly lower than for Tx, (entries 2 and 3 Table 3), indicating that both oxazines have good antioxidant capacity. Nevertheless, no parameters could be determined by the DPPH^●^ technique, due to delayed response of these oxazines.

In addition, the electrochemical behavior was evaluated trough cyclic voltammetry experiments. The results obtained from these experiments were anodic and cathodic peak potentials (E_ap_ and E_cp_, respectively) and the ∆E_p_ value. Tx exhibit the lowest E_ap_ value (0.123 V) and smallest ∆E_p_ (0.076 V) (entry 1 Table 3) while HQSAc presents E_ap_ and ∆E_p_ values of 0.367 and 0.273 V, respectively (entry 5 Table 3). However, oxazines **1** and **2** have oxidation potentials were higher than Tx and HQSAc, and a ∆E_p_ close to 1 V (entries 2 and 3, as seen in Table 3). These results indicate that the glassy carbon working electrode surface is less reactive for the heterogeneous redox processes of oxazine 1 as well as 2, presenting a kind of limitation in comparison with those of the reference compounds.

All these results are in agreement with the ARP values obtained from the DPPH^●^ tests (where the reaction response increases in the same order: oxazines << HQSAc < AAc < Tx). These results are also supported by the evidence previously reported [48,49,50], where the reaction with DPPH^●^ behaves like an ET reaction, instead of the typical HAT mechanism, under our reaction conditions.

In summary, this study demonstrated the in vitro antioxidant capacity of two derivatives of oxazine (compounds **1** and **2**), at concentrations of 9.0 µg/mL, 13.5 µg/mL, 18.0 µg/mL, 22.5 µg/mL and 27.0 µg/mL by the test of inhibition of OH^●^, ABTS^●+^ and AChE. The antioxidant capacity was confirmed ex vivo by the test of inhibition of hemolysis induced by AAPH in rat erythrocytes. However, these compounds have a limited response to some assays as it was demonstrated through cyclic voltammetry experiments and DPPH assays.

**Scheme 1:**
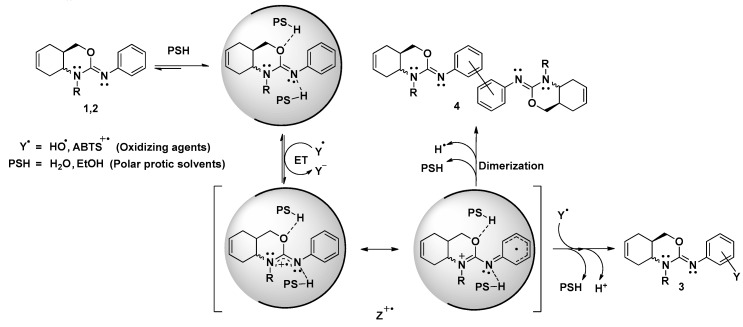
Reaction mechanism proposed for the radicals inhibition via ET by oxazines 1,2 in polar protic solvents. PSH: Polar protic solvents; EtOH: ethanol; ET: Electron Transfer process; Z^●+^: Radical cation intermediate.

In addition, although the final products of the redox reaction of oxazines 1 and 2 have not been elucidated, a tentative radical inhibition mechanism is provided in the Scheme 1 based in our experimental results. As it is possible to see, when the oxidation reaction is performed in polar protic solvents, the DPPH^●^ is unable to react by both a HAT, due to steric reasons, or ET for having a high oxidation potential, near to 1V. However, when oxazines are exposed to react with radical species like ABTS^●+^ or HO^●^, the radical inhibition reaction occurs via ET, being the imine moiety (>C=N-Ph) oxidized. In this way, the radical cation (Z^●+^) is formed and under go through a dimerization reaction (with one Y^●^ reduced by oxazine) or a complexation reaction (with a stoichiometry 2:1), yielding 3 and 4 as final products.

It must be emphasized that in the case of ABTS^●+^ the stoichiometric values do not completely explain the proposed mechanism. If the reaction takes place only through the mechanism with Y^●^ coupling path as last step, the number of ABTS^●+^ reduced should be 2. Nevertheless, the number of radical reduced are 1.1 and 1.5 for oxazines 1 and 2, respectively (entries 2 and 3 Table 3). This fact probably indicates that oxazine 1 has principally dimerization reaction as last step while oxazine 2 appears to follow the two different end pathways in Scheme 1. To support our hypotheses, it would be interesting to characterize the reaction products using liquid or gas chromatograph coupled with a mass spectrometer.

## 5. Conclusions

In summary, in vitro and ex vivo methods demonstrated that cyclohexene-fused 1,3-oxazines 1 and 2 have a potential antioxidant capacity compared with the classical reference compounds. This is an initial screening for determining the pharmacological potential of these substances as sources of active compounds with antioxidant properties.

Despite the fact that the mechanism and the antioxidant potential of these substances were elucidated with the help of different techniques, further studies should be performed to generalize the activities observed. In this sense, new chemical, pharmacological and biotechnological studies are needed in order to support the feasibility of using these substances for quenching free radicals and to obtain detailed structure-activity relationships for these compounds.

## Figures and Tables

**Table 1:** Antioxidant capacity of compounds **1**, **2** and Tx against hydroxyl radical (OH^●^) radical.

Compound	Antioxidant Capacity (%)				
	9.0 μg mL^−1^	13.5 μg mL^−1^	18.0 μg mL^−1^	22.5 μg mL^−1^	27.0 μg mL^−1^
**1**	71 ± 3	76.4 ± 0.2	76.9 ± 0.1	77.8 ± 0.1	78.5 ± 0.3
**2**	69.1 ± 0.3	79.2 ± 0.1	79.2 ± 0.3	79.4 ± 0.2	79.6 ± 0.1
Trolox	24 ± 1	40.3 ± 0.3	60 ± 5	62 ± 6	82 ± 2

**Table 2:** Antioxidant capacity of compounds **1**, **2** and Tx against OH^●^ radical.

Compound	Antioxidant Capacity (%)				
	9.0 μg mL^−1^	13.5 μg mL^−1^	18.0 μg mL^−1^	22.5 μg mL^−1^	27.0 μg mL^−1^
**1**	32.0 ± 0.4	36.4 ± 1	39.8 ± 0.4	42 ± 1	40.8 ± 0.5
**2**	38 ± 3	46 ± 4	46 ± 2	46 ± 1	46.6 ± 0.2
Trolox	38 ± 1	36.5 ± 0.5	37.7 ± 0.3	41.0 ± 0.6	41.3 ± 0.6

**Table 3:** Spectroscopic and Voltammetric analysis of different compounds.

Entry	Compound	Spectroscopic Analysis						Voltammetric Analysis		
		DPPH^●^			ABTS^●+^			CV		
		rEC_50_	ARP	N° of reduced DPPH^●^	rEC_50_	ARP	N° of reduced ABTS^●+^	E_ap_ (V)	E_cp_ (V)	∆Ep(V) = |Ecp − Eap|
1	Trolox	0.204	4.9	2.4	0.30	3.3	1.6	0.123	0.047	0.076
2	**1**	0	0	0	0.45	2.2	1.1	0.420	−0.559	0.979
3	**2**	0	0	0	0.34	2.9	1.5	0.380	−0.559	0.939
4	AAc ^a^	0.227	4.4	2.2	-- ^b^	-- ^b^	-- ^b^	0.322	-- ^c^	-- ^c^
5	HQSAc ^a^	0.279	3.6	1.8	-- ^b^	-- ^b^	-- ^b^	0.367	−0.094	0.273

^a^ These experiments were performed in order to check the negative results obtained with the oxazines **1** and **2**. ^b^ No ABTS^●+^ tests were performed with AAc and HQSAc. ^c^ None reversible process was observed. CV: cyclic voltammetry; E_ap_: anodic peak potential; E_cp_: cathodic peak potential.
